# Enhancing cognitive control of our decisions: Making the most of humor during the IGT in females and males

**DOI:** 10.3758/s13415-024-01210-y

**Published:** 2024-09-05

**Authors:** Jorge Flores-Torres, Kateri McRae, German Campos-Arteaga, Lydia Gómez-Pérez

**Affiliations:** 1 Escuela de Psicología, Pontificia Universidad Católica de Chile, Santiago, Chile; 2 Laboratorio Neurociencias, Escuela de Medicina, Pontificia Universidad Católica de Chile, Santiago, Chile; 3 Department of Psychology, University of Denver, Denver, CO USA; 4 Escuela de Psicología, Universidad Tecnológica Metropolitana, Santiago, Chile; 5 Departamento de Personalidad Evaluación y Tratamiento Psicológico, Facultad de Psicología y Logopedia, Universidad de Málaga, Málaga, Spain

**Keywords:** Humor, Iowa Gambling Task, Decision-making, Gender, Cognitive control

## Abstract

We studied the impact of humor on the Iowa Gambling Task (IGT) decision-making performance and the cognitive control exerted during this task, considering sex as a moderator, and examined whether cognitive control mediated the influence of humor on decision-making. Sixty participants (30 females) performed an extended version of the IGT (500 trials divided into 20 blocks). We randomly assigned them to either an experimental group (Humor Group; Hg; *n* = 30), where humorous videos were interspersed in the decision-making trials or a control group (Non-Humor Group; NHg; *n* = 30), where nonhumorous videos were interspersed in the decision-making trials. We recorded participant performance and feedback-related negativity (FRN) and P3b event-related potentials (ERP) during IGT feedback as task monitoring and attention allocation indicators, respectively. We expected that whereas humor would improve IGT decision-making under risk in females during the last blocks (17–20) as well as cognitive control (specifically attention allocation and task monitoring) across the entire IGT, it would impair them in males. Contrary to our expectations, humor improved IGT decision-making under risk for both sexes (specifically at blocks 19 and 20) and attention allocation for most IGT blocks (P3b amplitudes). However, humor impaired IGT decision-making under ambiguity in males during the block six and task monitoring (FRN amplitudes) for most IGT blocks. Attention allocation did not mediate the beneficial effect of humor on decision-making under risk in either sex. Task monitoring decrements fully mediated the humor's detrimental influence on men's decision-making under ambiguity during block six.

**Supplementary Information:**

The online version contains supplementary material available at 10.3758/s13415-024-01210-y.

## Introduction

The Iowa Gambling Task (IGT; Bechara et al., 1994) captures the intricate interplay between emotions and cognition in real-life decision-making. Originally developed to assess decision-making impairments in individuals with prefrontal cortex damage (Bechara, 2005), the IGT has been employed to study impulsivity and compromised decision-making in conditions, such as substance abuse and pathological gambling (Kovács et al., 2017). It also has gained widespread use in research with nonclinical participants, revealing significant associations with psychological factors, such as reward responsiveness, fun-seeking, impulsivity, and physiological markers linked to inhibitory control (Giustiniani et al., 2019; Suhr & Tsanadis, 2007).

Participants playing the IGT face four card decks and try to maximize gains while minimizing losses without any a priori information. Each deck represents different reward-to-punishment ratios, which participants must learn as they play. According to the Somatic Marker hypothesis (Bechara, 2005), both emotional and cognitive processes are needed to achieve superior performance; however, what these processes exactly mean has been substantially challenged across time (Bowman et al., 2005; Fernie & Tunney, 2013). Brevers et al. (2013) updated terms into hot and cool executive processes. Thus, during early blocks^1^ people require using hot processing, because the probabilities are unknown and cannot be estimated rationally, so the decision-making is to some extent emotionally guided (decision-making under ambiguity). By contrast, the last block operates under a cool executive control, because at that point probabilities are known and rationally guided (decision-making under risk). During hot processing, participants engage in exploring various deck options to grasp the associations between decks and the reward and punishment outcomes (i.e., the emotional processing of monetary feedback; Bechara, 2005; van den Bos et al., 2012). Players first prioritize immediate substantial rewards, often disregarding the risk of accumulating losses. Near the end of the task, successful participants depart from this high-risk default strategy and settle into making choices that lead to smaller gains but also result in smaller losses. As a result, we see a transition from ambiguity to decision-making under risk; the task depends more on cool executive function, because the deck’s probabilities are known (Brevers et al., 2013).

Growing research highlights sex's crucial role in untangling the intricate interplay between emotion and cognition^2^ (Bolla et al., 2004; Flores-Torres et al., 2019, 2022; Overman et al., 2006; Reavis & Overman, 2001; Weller et al., 2010). While the influence of cultural and social contexts should not be dismissed, consistent findings emphasize fundamental processes that deserve attention. For instance, females typically manifest higher emotional intensity and responsiveness to negative valence stimuli compared with males (Van Hasselt et al., 2012; Overman et al., 2011; Flores-Torres et al., 2022). Moreover, sex differences in brain activation during cognitive reappraisal indicate that females recruit prefrontal resources typically indicative of cognitive control to a greater extent than males, while simultaneously recruiting less activation in regions associated with reward (McRae et al., 2008). Therefore, there is reason to predict that the interaction between positive emotion and cognitive control is moderated by sex. However, our previous IGT study collected only task performance, so the neural processes engaged during this interaction remained unclear.

Considering the factors mentioned, it is not surprising that consistent evidence supports sex differences on IGT performance (Bolla et al., 2004; Flores-Torres et al., 2019, 2022; Overman et al., 2006; Reavis & Overman, 2001; Weller et al., 2010). Women typically earn less money and take more trials to select consistently advantageous decks with long-term benefits compared with men (Bolla et al., 2004; Flores-Torres et al., 2019; Flores-Torres et al., 2022; Weller et al., 2010). These sex differences primarily emerge during the second half of the task, indicating that they are related to maintaining (cool) cognitive control rather than solely the (hot) learning of the task.

Cognitive control is a neural mechanism crucial for goal-directed behaviors, which enables us to engage in complex behaviors designed to achieve goals by overriding or modifying automatic and habitual responses (Mäki-Marttunen et al., 2019; Miller, 2000). Notably, cognitive control is not only involved during IGT performance but also during humor processing. The relationship between humor and cognitive control is clear during the cognitive processes involved in creating, understanding, and responding to humor. For instance, humor arises from incongruities, surprises, or deviations from expectations, such as when we understand something that could be interpreted in different ways and purposedly we choose to play with those meanings or by inhibiting the expression of something not funny (Beaty & Silvia, 2012; Martin, 2007; Zabelina & Ganis, 2018). Given that cognitive control is necessary for both solving the IGT (Bolla et al., 2004; Flores-Torres et al., 2019; Flores-Torres et al., 2022; Weller et al., 2010) and processing humor (Beaty & Silvia, 2012; Zabelina & Ganis, 2018), it is conceivable that participants who engage in alternating tasks involving humorous stimuli and the IGT might experience a competition for cognitive resources. Consequently, this competition could lead to a decline in performance on the IGT. However, it is important to note that this assertion assumes that men and women process humor and the IGT in a similar manner, which is not the case.

First, functional neuroanatomical sex differences in humor processing have been observed, with greater left prefrontal cortex activation in females when exposed to humorous stimuli compared with males (Azim et al., 2005). Another study found that females tend to use an emotional/limbic pathway while processing humor, whereas males rely more on a cognitive/executive pathway (Kohn et al., 2011). Second, sex differences in neuroanatomy and function also have been associated with variations in performance on the IGT. Females have shown hypoactivity in limbic structures related to affective and reward control, as well as lower emotional control compared with males (Van Hasselt et al., 2012; Overman et al., 2011; Bechara, 2005; van den Bos et al., 2012). These findings indicate significant sex differences in the mechanisms of cognitive and emotional control. Evidence suggests that females may achieve better cognitive control through emotion regulation, whereas males may achieve cognitive control directly without depending on emotion regulation (Flores-Torres et al., 2022; Van Hasselt et al., 2012; Overman et al., 2011; Kohn et al., 2011). However, further research in this regard is still needed.

In a previous study, we investigated the impact of humor on decision-making during the IGT, considering the moderating effect of sex (Flores-Torres et al., 2019). Participants were exposed to either brief humorous or nonhumorous videos before each IGT decision. One-half of the sample was exposed to 100 brief humorous videos (humor group, Hg); the other half was exposed to 100 brief nonhumorous videos (nonhumor group, NHg)—see supplementary material. Women in the Hg demonstrated higher IGT performance than women in the NHg in the last block of the task, suggesting that humor had a beneficial effect on females’ IGT performance; by contrast, males in the Hg demonstrated lower IGT performance than males in the NHg in the last block of the task, indicating that humor impaired males IGT performance.

As far as we know, our previous study remains the only investigation that has delved into the intricate relationship among humor, sex, and decision-making on the IGT. Given this unique position, it is essential to replicate our findings to ensure their validity. Furthermore, while cognitive control may explain, at least partially, the effect of humor in decision-making, to our knowledge, no studies measuring neural correlates of cognitive control have been conducted yet. Therefore, this research has three main objectives. First, we attempt to reevaluate how humor and sex influence decision-making with a new sample. Second, we intend to explore how humor and sex impact the EEG activity associated with cognitive control during IGT performance as it evolves over time. Our third objective is to explore whether cognitive control mediates the influence of humor on decision-making and to explore whether this relationship also is moderated by sex.

Cognitive control has been linked to several event-related potentials (ERPs). Although names and functional descriptions differ, a rather uniform sequence of ERPs is found during the IGT: an early frontocentral negativity named feedback-related negativity (FRN), which is especially relevant during the beginning of the task (Bianchin & Angrilli, 2011; Chandrakumar et al., 2018; Cohen et al., 2007; Holroyd et al., 2005) and a later positive deflection mostly at parietal sites—named P3b—which is especially informative late in the task (Kok, 2001; Polich, 2007). The FRN component is expected, especially after punishments, and is interpreted as a signal indicating incoming evidence for the potential necessity of action adaptation (Wetzels et al., 2010; Ullsperger et al., 2010, Steinhauser & Yeung, 2010). As the evidence of past choices accumulates, a later posterior positivity appears—P3b—which is considered a biomarker of the amount of attention available for task performance (Kahneman, 1973; O’Connell & Hofmann, 2012). In the context of the IGT, P3b amplitude is an index of attentional resource allocation to process cognitive aspects of the task feedback, which is fundamental to solve the task (Turetsky et al., 2009; O’Connell & Hofmann, 2012; Polich, 2007). Therefore, we assessed these two ERPs components—FRN and P3b during reward and punishments—as indicators of cognitive control. The first of these components will inform us about task learning, that is, how participants use the information provided through feedback to correct and improve their future decisions. The second one will inform us about attentional resource allocation toward IGT performance improvement.

Participants were exposed to either brief humorous or nonhumorous videos during the IGT. Building on previous research, we predicted that men in the humor group (Hg) would exhibit lower IGT performance during the final blocks (decision-making under risk), specifically from block 17 to 20, compared with men in the nonhumor group (NHg). Men in the Hg were anticipated to have reduced amplitudes in the EEG correlates of cognitive control (FRN and P3b) across the entire task compared to the men in the NHg. By contrast, we expected that women in the Hg would exhibit higher IGT performance, especially during the final blocks (17–20), compared with women in the NHg. We anticipated that women in the Hg would exhibit increased amplitudes in FRN and the P3b components throughout the task, in contrast to the women in the NHg. Lastly, we hypothesized that cognitive control would mediate the relationship between humor and IGT performance and tested whether this was moderated by sex.

## Materials and methods

### Participants

Inclusion criteria for participation were (1) being an undergraduate student, (2) speaking Spanish, and (3) having normal or corrected-to-normal vision. Exclusion criteria were (1) reporting current diagnosis of a neuropsychiatric disorder, (2) scores on the Self-Report Questionnaire (SRQ) (Harding et al., 1980; Vielma et al., 1994) indicating severe depressive symptomatology, (3) reporting a history of drug or alcohol abuse and/or consumption of drugs within 24 h of participating in the experimental task.

The sample size was determined before data collection, based on our previous study (Flores-Torres et al., 2019). According to analyses performed with the software G* Power 3.1, in that study for a sample of 68 participants [34 women (17 experimental and 17 control) and 34 men (17 experimental and 17 control)], we obtained a large effect size of Fs (0.47), and a statistical power of 0.97 using a probability error α (0.05) for hypothesized differences in the interaction (Sex by Condition by Blocks). Because we are using the same experimental design, we calculated the sample size for the present study for a large effect size Fs (0.40), a probability error α (0.05), and a statistical power of (0.80), which leads to 60 participants (30 women and 30 men). Finally, 64 participants (33 women and 31 men) completed the study. Data from four of these participants (1 man and 3 women) were not included in the analyses, because it was determined after participating that they did not fulfill participation criteria. One of the participants reported having consumed drugs before the experiment, and three of them presented severe depressive symptomatology according to the SRQ. Therefore, data from 60 participants (30 men; mean age 23.05, *SD* = 4.6 and 30 women; mean age 23.6, *SD* = 4.4) were included in the final analyses.

### Questionnaires and instruments

#### The Self-Report Questionnaire (Harding et al., 1980)

The SRQ was used to assess depressive symptomatology. It consists of 25 yes/no questions. The SRQ has been validated for the Chilean population (Vielma et al., 1994). Subjects answering affirmatively to questions 21 to 25 (elevated probability of depressive symptomatology) were not included in the study sample, because depression has been shown to affect decision-making (Battersby et al., 2006; Dalgleish et al., 2004).

#### The State-Trait Anxiety Inventory (STAI) (Spielberger, 1970)

The STAI was used to assess anxiety symptoms. It consists of 40 questions divided into two subscales: state anxiety and trait anxiety. The STAI has been validated for the Chilean population (Vera-Villarroel et al., 2007). We assessed this construct because higher trait anxiety scores have been previously associated with impairments in decision-making and could potentially affect our results (Miu et al., 2008).

#### Humorous and nonhumorous videos

We used a selection of 200 videos (100 humorous and 100 nonhumorous). These videos were used in our previous study, in which they were rated as equally funny by women and men. For a detailed description, see supplementary material and Flores-Torres et al., 2019.

#### The IGT

The IGT was designed as a realistic decision-making task (Bechara et al., 1994; Hooper et al., 2004). On each trial, participants choose a card from one of four card decks (A, B, C, and D). After each choice, participants might be rewarded with either virtual money (reward) or punished with a loss of virtual money (punishment). Participants must learn as they play which are the advantageous and disadvantageous decks to solve the task and maximize earnings. Participants can change decks at will; however, they are warned that some decks are worse than others in terms of total payment and that the win/loss proportions and amounts stay fixed within each deck. Likewise, they are informed that the goal is to win as much money as they can or to avoid losing money as much as possible.

Card decks A and B are monetarily risky/disadvantageous, and C and D are monetarily safe/advantageous. Card decks A and B are associated with large, immediate rewards (e.g., $100), but continuing to select from these decks results in accumulating less profit, or loss, because of occasional, large monetary punishments. Choosing from card decks A and B leads to a net loss of $250 during the first 10 trials. By contrast, card decks C and D are associated with small immediate rewards (e.g., $50) but with small monetary punishments. Continuing to select from these decks results in accumulating more profit, and choosing from decks C and D leads to a net gain of $250 during the first 10 trials.

For the present research, to measure reliably the event-related potentials (ERP), we modified the original IGT following Cui et al. guidelines (2013). Namely, we increased the number of trials from 100 to 500 and modified the feedback shown to participants, such that the presentation of win and losses observed in the original task was replaced by net scores. According to previous studies (Flores-Torres et al., 2019, 2022; Weller et al., 2010), we expected to find differences in the last blocks for the present study, blocks 17 to 20.

#### EEG data acquisition and preprocessing

EEG data were recorded on a Biosemi Active two system. We used 64 active electrodes placed according to the international 10/20 extended system. Horizontal and vertical eye movements were monitored using four external electrodes. Horizontal EOG was recorded bipolarly from the outer canthi of both eyes and vertical EOG was recorded from above and below of the participant’s right eye. Data pre-processing was performed by using MATLAB v8.3.0.532 (Matlab, 2014) with EEGLAB v13.6.5b toolbox (Delorme & Makeig, 2004) and ERPLAB v9.20 toolbox (Lopez-Calderon & Luck, 2014). Following Cui et al.’s guidelines (2013), the signal was down-sampled offline at 512 Hz, and all electrodes were referenced to averaged mastoids. Following Tanner et al.’s guidelines (2016), a second-order infinite impulse response Butterworth filter was used for band-pass filtering continuous EEG data, with a half amplitude cutoff frequency of 0.1 Hz and 30 Hz. For channels and artifact rejection, we performed a semiautomated channel rejection coupled with visual inspection applying EEGLAB “trim outlier” function. We set a high upper bound rejection threshold of 200 μV. The total average of rejected channels was 0.6 channels per subject. Commonly recorded artifactual potentials (CRAP: Luck, 2014) detection was performed by setting a high rejection criterion (± 100 μV) threshold for the moving window peak-to-peak algorithm. This procedure allowed rejecting extremely noisy data, which never exceeded 15% of total trials (75/500 trials). We also performed visual inspection to detect muscle-like activity. Then, we performed independent component analysis (ICA; Delorme & Makeig, 2004) decomposition on continuous data, through infomax RunICA algorithm, to eliminate an average of two ocular stereotypical artifactual components per EEG recording. RunICA is an iterative algorithm that minimizes the non-Gaussianity of the components, which allows for its separation and, in our case, facilitates the subsequent identification of artifactual components. Then, we performed a channel spherical interpolation function.

#### Electrophysiological assessment

The EEG signal was segmented into 500 trials. For each trial, we analyzed the 200 ms immediately prior to feedback until 1,000 ms after feedback onset. The average number of epochs after artifactual rejection and correction was 460 (*SD* = 10). The prestimulus window of 200 ms was used to correct baseline activity in each trial. Epochs were averaged for each subject, and then averaged by sex (males vs. females), condition (humor vs. nonhumor), feedback (reward vs. punishment), and Blocks (Block 1 [trials 1–100], Block 2 [trials 101–200], Block 3 [trials 201–300]), Block 4 [trials 301–400], and Block 5 [trials 401–500], based in Garrido-Chaves et al. (2021). We have chosen to work with five blocks of 100 trials each, rather than 20 blocks with 25 trials each, mirroring the original segmentation performed in Bechara’s behavioral analyses. This decision is based on recent studies on ERPs where in between-group designs, statistical power doubles when transitioning from 45 to 90 trials, even when having just 12 subjects per condition (Fischer et al., 2017; Boudewyn et al., 2018).

For tmax permutation analysis (Groppe et al., 2011a, 2011b) (see *Data Analyses*), the data were down-sampled to 128 Hz to decrease the number of comparisons and increase statistical power (Luck, 2014). Following an examination of the permutation results, mean difference wave for the FRN amplitude was set within 100–250-ms time window relative to the 200-ms prestimulus baseline and computed for the three electrodes of interest (FZ, FCZ and CZ), selection based on existing literature (Chandrakumar et al., 2018; Cohen et al., 2007). Cluster mass permutation test (Blair & Karniski, 1993) procedure was performed for the P3b amplitudes, which was set within 350–450-ms time window relative to the 200-ms prestimulus baseline and computed for 36 electrodes of interest: FP1, AF7, F7, F5, FT7, C1, C3, C5, T7, CP1, CP3, TP7, P1, P3, P5, PO3, P9, FCZ, CZ, CPZ, PZ, POZ, OZ, C2, C4, T8, CP2, CP4, CP6, TP8, P2, P4, P6, P8, PO4, PO8. This resulted in a matrix of widely distributed electrodes for P3b activity derived from actual data, rather than relying on a preselected electrode matrix for P3b analysis, as shown in previous studies (Cui et al., 2013).

### Procedure

Our research protocol was approved by the Ethics Committee of the Pontificia Universidad Católica de Chile (PUC). Participants provided written consent in accordance with the Declaration of Helsinki. All experiments were performed at the Neuro-dynamic Laboratory of the School of Psychology of the PUC. We recruited participants for the study through an advertisement published on the PUC student website. Those interested in participation were informed about the inclusion and exclusion criteria and provided with more study details via email. If they reported that they met the inclusion criteria, we invited them to come to the lab. Participants were given a movie ticket as compensation for their time used in the laboratory.

In-lab session. First, we provided participants with more details about the study and completed the informed consent process. Next, participants completed the SRQ and the STAI-t. Then, they sat down in a comfortable chair in front of a computer screen, and the electrodes were connected. After the electrode application was completed, participants started the experimental task. Task instructions were presented on the computer screen. The distance from participant’s eyes to the computer screen was 60 cm, visual angle 4.7°, refresh rate 144 frames per seconds. Study duration was approximately 1 h.

Each trial began with the word “*video,*” which appeared on the screen for 1,500 ms. Then, the video itself appeared (5,000 ± 1,000 ms of duration), followed by five decision-making trials. Because participants made five decisions for each video, by the end of the task, each participant completed 100 different videos and 500 IGT trials. During these trials, participants saw four deck options (labeled A, B, C, and D) and chose one by left clicking on their preference with a USB mouse. When participants selected a deck, its perimeter lit up in red. After that, the screen changed to black for 300 ms, which allowed for a clean baseline for ERP measurement. Then, feedback appeared for 2,000 ms, the onset of which corresponds to “0” time to evaluate ERPs. Feedback could be a net gain (e.g., + 100) or a net loss (e.g., − 50). Each card’s feedback depended on the probabilities according to the Bechara IGT manual (2007), using modifications for an electrophysiological adaptation (Cui et al., 2013). During the screen showing the four deck options, on the central superior area of the screen, two bars appeared. A green bar showed cumulative wins and losses and a red bar represented the amount of money they owed (all participants started the task with $2,000 CLP of virtual money [around $3 USD]). After feedback, these bars automatically updated according to the feedback on that trial. We emphasized to participants that positions and deck contingencies were fixed during the whole task, that they could change decks at will, and that there was no association whatsoever between the videos and the decks. Participants had no specific information about how to solve the task, nor did they know how long it would take. Participants completed 100 videos and 500 IGT trials (divided into 20 blocks of 25 trials each). We programmed two breaks (after 40% and 70% of total trials). As mentioned, we calculated IGT performance every 25 trials. We calculated individual block performance scores and not cumulative total ones, whereas we calculated mean FRN and P3b amplitudes every 100 trials. Therefore, we have five measurements of FRN and P3b and 20 measurements of IGT performance throughout the experiment.

### Data analyses

To test our behavioral hypotheses, we conducted several three-way mixed ANOVAs and MANOVAS. Before them, we checked normality, linearity, and sphericity assumptions. We used the parameter ε Greenhouse–Geisser to correct for sphericity violations. Our significance threshold was *p* < 0.05, with Bonferroni corrections for post-hoc comparisons. Outliers were replaced using the mean plus two standard deviations method recommended by Field (2013).

We corrected for multiple comparisons by using sequential Bonferroni-Holm and Benjamini–Hochberg procedures (Cramer et al., 2016). To test our neurophysiological ERPs, we performed permutation tests based on tmax statistics (Blair & Karniski, 1993). One critical advantage of the tmax procedure is that it uses the characteristics of the actual data to evaluate statistical significance. This method was chosen, because it allows to determine, with strong confidence and without previous assumptions, the temporal dynamic and topographical distribution of an effect, controlling adequately for Type-1 and Type-2 error (Luck, 2014). We performed tmax permutation procedures on mean difference wave amplitudes for a global time window. We did this for FRN (100–250 ms) on three representative midline electrodes (FZ, FCZ, and CZ). For the P3b amplitude, we observed that previous studies have used an a priori highly distributed selection of electrodes (Cui et al., 2013). Thus, we preferred using a cluster mass permutation test to determine both topography and time distribution based on the actual data.

The cluster-based permutation tests operate by building a null hypothesis distribution from the most extreme statistic from each time point/electrode. First, it forms clusters of neighboring extreme *t*-scores and then builds the null hypothesis distribution from the most extreme cluster statistic (e.g., the sum of all the *t*-scores in the cluster).

The results for cluster mass permutation were set within 350–450-ms time window relative to the 200-ms prestimulus baseline and determined 36 electrodes of interest: FP1, AF7, F7, F5, FT7, C1, C3, C5, T7, CP1, CP3, TP7, P1, P3, P5, PO3, P9, FCZ, CZ, CPZ, PZ, POZ, OZ, C2, C4, T8, CP2, CP4, CP6, TP8, P2, P4, P6, P8, PO4, PO8.

To examine whether the effect of humor on IGT performance was mediated by cognitive control and whether this mediational effect was moderated by sex, we conducted several path analyses with standard errors, indirect effects, and nonsymmetric 3,000 bootstrap confidence intervals (CI) by using the software MPlus (Muthén & Muthén, 2017). We employed the mean cumulative scores of targeted IGT block performance and mean cognitive control scores (averaged amplitudes for FRN and P3b components), both transformed to *z*-scores, to construct comparable mediational models across multiple timepoints.

## Results

### Preliminary analyses

Because trait anxiety has been shown to affect IGT performance, and women report more trait anxiety than men (Miu et al., 2008), we examined differences in this variable between the groups. Namely, we conducted a two-way between subject factorial ANOVA (Sex [male and female] by Group [humor and nonhumor]), in which the dependent variable was trait anxiety. The main effect of Sex, the main effect of Group, and the interaction were not statistically significant, indicating that there were no significant differences in trait anxiety among groups. Therefore, we did not consider this variable in further analyses.

### Differences in IGT Performance

**Fig. 1:**
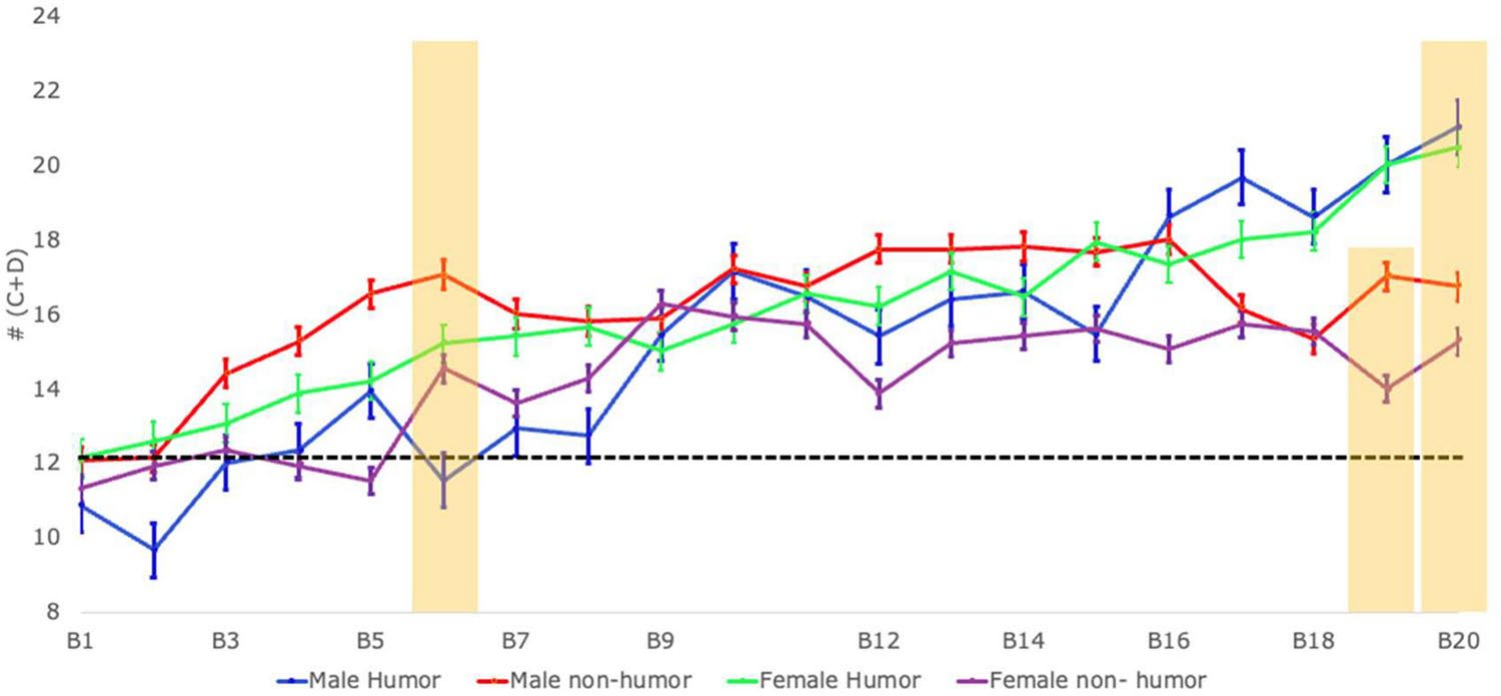
Results for the Iowa gambling task (IGT) performance, specifically the number of advantageous decks chosen across 20 blocks of 25 trials each. The plot shows performance by sex, group, and blocks, the black dotted line indicates the threshold for random (lower than 12) vs. nonrandom choices (above 12). Error bars indicate the standard error of the mean, and yellow bars indicate blocks with statistically significant results

To examine the effect of humor on IGT performance and whether the effect of humor differed by sex, we conducted a three-way repeated measures ANOVA (sex [male and female] by group [humor and non-humor] by blocks [from 1 to 20]) considering as the dependent variable the number of advantageous decks chosen (C + D). Results revealed a significant main effect of blocks *F* (3,132) = 3.83, *p* < 0.01; *η*_*p*_^*2*^ = 0.08, 95% CI [0.31, 0.69] indicating that performance improved across the task. The blocks by group interaction was statistically significant (*F* (19,38) = 2.57, *p* < 0.01; *η*_*p*_^*2*^ = 0.56, 95% CI [0.06, 0.54]), indicating that participants in the Hg exhibited higher IGT performance during the end of the task, during block 19 (trials 451–475; *t* = 4.46, *p* = 0.01) and block 20 (trials 476–500; *t* = 5.03, *p* < 0.01) than participants in the NHg (See Fig. 1). The triple interaction of sex by group by blocks was statistically significant *F* (19,38) = 1.88, *p* = 0.04; *η*_*p*_^*2*^ = 0.48, 95% CI [0, 0.45]. Specifically, our analysis revealed that males in the Hg initially exhibited lower IGT performance than males in the NHg during block six (trials 126–150; *t* = -5.53, *p* < 0.01, 95% CI -9.47, -1.6). Nevertheless, this last result must be interpreted with caution since the triple interaction of sex, group and block did not survive our correction procedures, meaning it may not be reliable. Preserving specially the main effect of blocks and the interaction blocks by group (See Table 1).

**Table 1:** Results from the sequential Bonferroni (seqB) and Benjamini-Hochberg (BH) procedures for a three-way factorial analysis (sex x group x blocks) on IGT performance

Effect	*p*	α_adj_SeqB	α_adj_SeqBH	*H0* seqB	*H0* seqBH
Blocks	.0001	.0071	.0071	rejected	Rejected
Blocks x Group	.006	.0083	.0142	rejected	Rejected
Blocks x Sex x Group	.04	.0100	.0214	retained	Retained
Blocks x Sex	.05	.0125	.0285	retained	Retained
Sex x Group	.3	.0166	.0357	retained	Retained
Sex	.6	.0250	.0428	retained	Retained
Group	.7	.0500	.0500	retained	Retained

α_adj_SeqB = the adjusted alpha level with the sequential Bonferroni procedure; α_adj_SeqBH the adjusted alpha level with the Benjamini–Hochberg procedure; *H0* seqB = evaluation of the null hypothesis with the sequential Bonferroni procedure; *H0* seqBH = evaluation of the null hypothesis with the Benjamini–Hochberg procedure

### Differences in FRN

Given that the FRN component exhibits a well-determined EEG topography, we conducted a series of tmax permutation tests on key midline electrodes for assessing FRN (FZ, FCZ, and CZ). We applied an average difference waveform within the time interval of 100–250 ms (identified through tmax permutation analysis). Our analysis encompassed sex and group as intergroup factors, and type of feedback (monetary reward and monetary punishment) and EEG-blocks [E-block one (trials 1–100), E-block two (trials 101–200), E-block three (trials 201–300), E-block four (trials 301–400), and E-block five (trials 401–500)] as intraparticipant factors. A comprehensive summary of the outcomes of tmax permutation tests on FRN component disparities can be found in Table 2.

**Table 2:** Results for the *t*-max permutation test differences on IGT feedback (reward and punishment), for group (humor and nonhumor), sex (male and female), time window (100–250 ms), blocks (1–5), and electrodes (FZ, FCZ, and CZ)

Comparison	Type of feedback	Block	Trials IGT	Time window	Electrodes	*t*	*p*
MHg-MNHg	Reward	1	1–100	100–250	FZ, FCZ, and CZ	1.89	**[.008, .002]**
		2	101–200		FZ	1.94	**.02**
		3	201–300		None	0	.05 *(NS)*
		4	301–400		None	0	.06 *(NS)*
		5	401–500		FZ, FCZ, and CZ	1.93	**[.04, .01]**
	Punishment	1	1–100		None	0	.15 *(NS)*
		2	101–200		None	0	.06 *(NS)*
		3	201–300		FZ	1.98	**.02**
		4	301–400		FZ, FCZ, and CZ	1.95	**[.04, .007]**
		5	401–500		FZ	2.01	**.01**
FHg-FNHg	Reward	1	1–100	100–250	None	0	.14 *(NS)*
		2	101–200		None	0	.49 *(NS)*
		3	201–300		None	0	.50 *(NS)*
		4	301–400		None	0	.54 *(NS)*
		5	401–500		None	0	.33 *(NS)*
	Punishment	1	1–100		None	0	.51 *(NS)*
		2	101–200		None	0	.47 *(NS)*
		3	201–300		None	0	.23 *(NS)*
		4	301–400		None	0	.23 *(NS)*
		5	401–500		None	0	.47 *(NS)*
MHg-FHg	Reward	1	1–100	100–250	None	0	.11 *(NS)*
		2	101–200		FZ	1.86	**.03**
		3	201–300		FZ, FCZ, and CZ	1.88	**[.04, .007]**
		4	301–400		FZ, FCZ, and CZ	1.89	**[.03, .001]**
		5	401–500		FZ and FCZ	1.85	**[.03, .033]**
	Punishment	1	1–100		FZ, FCZ, and CZ	1.87	**[.04, .02]**
		2	101–200		None	0	.15 *(NS)*
		3	201–300		FZ	1.99	**.01**
		4	301–400		FZ, FCZ, and CZ	1.93	**[.03, .01]**
		5	401–500		FZ and FCZ	1.97	**[.04, .007]**
MNHg-FNHg	Reward	1	1–100	100–250	None	0	.43 *(NS)*
		2	101–200		None	0	.43 *(NS)*
		3	201–300		None	0	.32 *(NS)*
		4	301–400		None	0	.10 *(NS)*
		5	401–500		None	0	.18 *(NS)*
	Punishment	1	1–100		None	0	.35 *(NS)*
		2	101–200		None	0	.49 *(NS)*
		3	201–300		None	0	.12 *(NS)*
		4	301–400		None	0	.08 *(NS)*
		5	401–500		None	0	.20 *(NS)*

*NS* = not significant

Tmax permutation test results for FRN amplitude component during humor and nonhumor conditions, across sex, and in response to reward and punishment feedback are as follows (5,000 permutations): MHg = male in humor group; MNHg = male in nonhumor group; FHg = female in humor group; and WNHg = female in nonhumor group

Significant *t*-scores (*t*) and corrected *p*-values (*p*) within the [higher and lower *p* range value] range

When comparing males in the Hg to males in the NHg, significant differences emerged in the FRN component. Contrary to our expectations, males in the Hg displayed a significant decrease in FRN amplitude during reward feedback compared with males in the NHg. This reduction was evident in the E-block one (trials 1–100) across the three electrodes measured (FZ, FCZ, and CZ) and in E-block two (trials 101–200) at the FZ electrode. Furthermore, a significant decrease in FRN amplitude was observed in the E-block five (trials 401–500), impacting all measured electrodes. Critically, during punishment instances, a statistically significant decrease in FRN amplitude was observed in males in the Hg compared with males in the NHg. This effect manifested in E-block three (trials 201–300) at the FZ electrode, block four (trials 301–400) across all measured electrodes, and E-block five (trials 401–500), specifically at the FZ electrode.

**Fig. 2:**
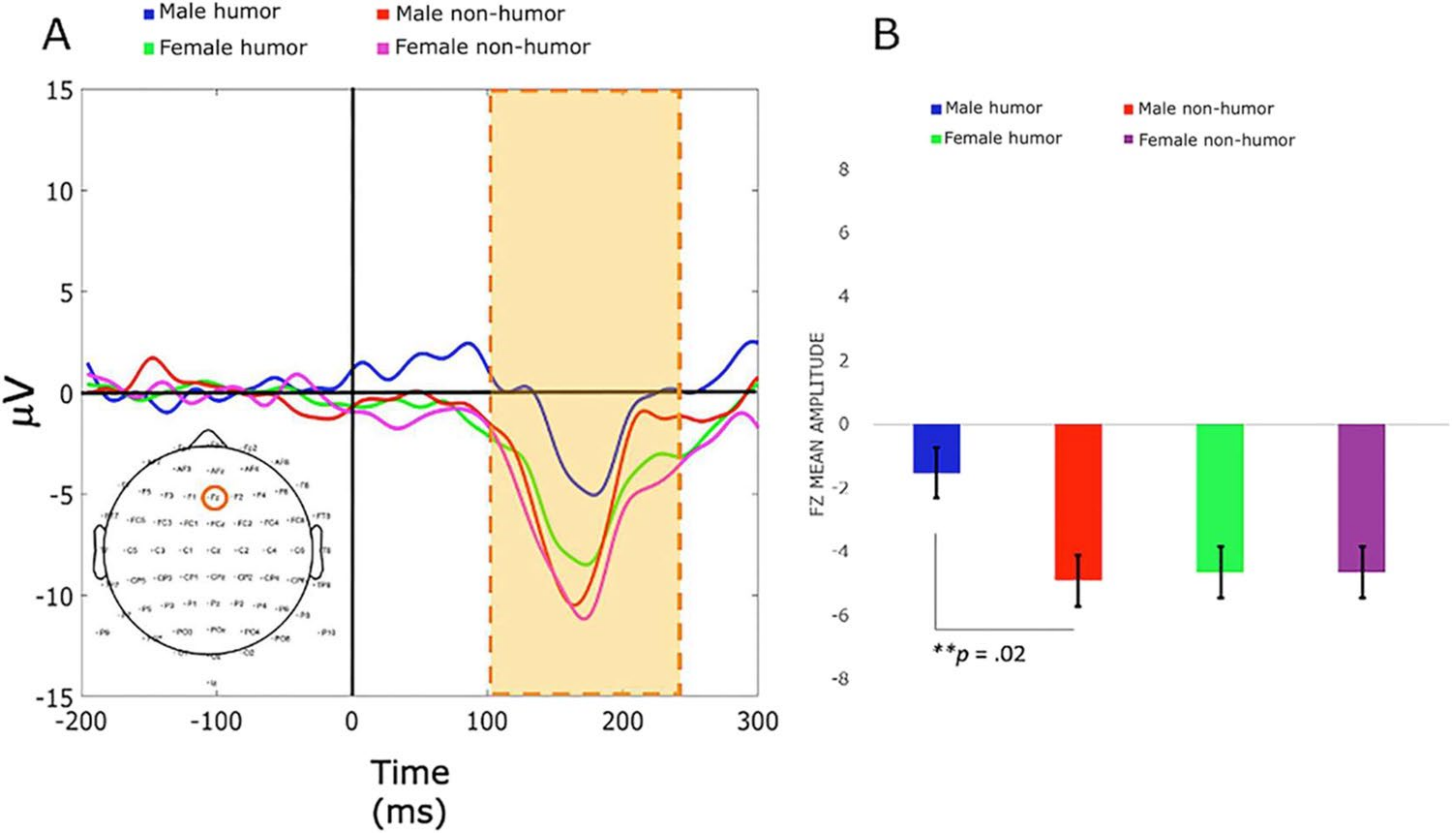
Grand-averaged ERP FRN waveforms from a representative electrode (central region FZ) after punishment feedback for all groups, during E-block four (IGT trials 301–400). Time window 100–250 ms. **A** Wave plot reveals statistically significant amplitude decreases for electrode FZ in males in the Hg compared with males in the NHg. **B** Bar plot for FZ amplitude mean shows a significant decrease in mean amplitudes for males in the Hg compared with males in the NHg. Error bars represent the standard error of the mean (SEM), and the yellow bar indicates statistically significant results within the time window measured

**Fig. 3:**
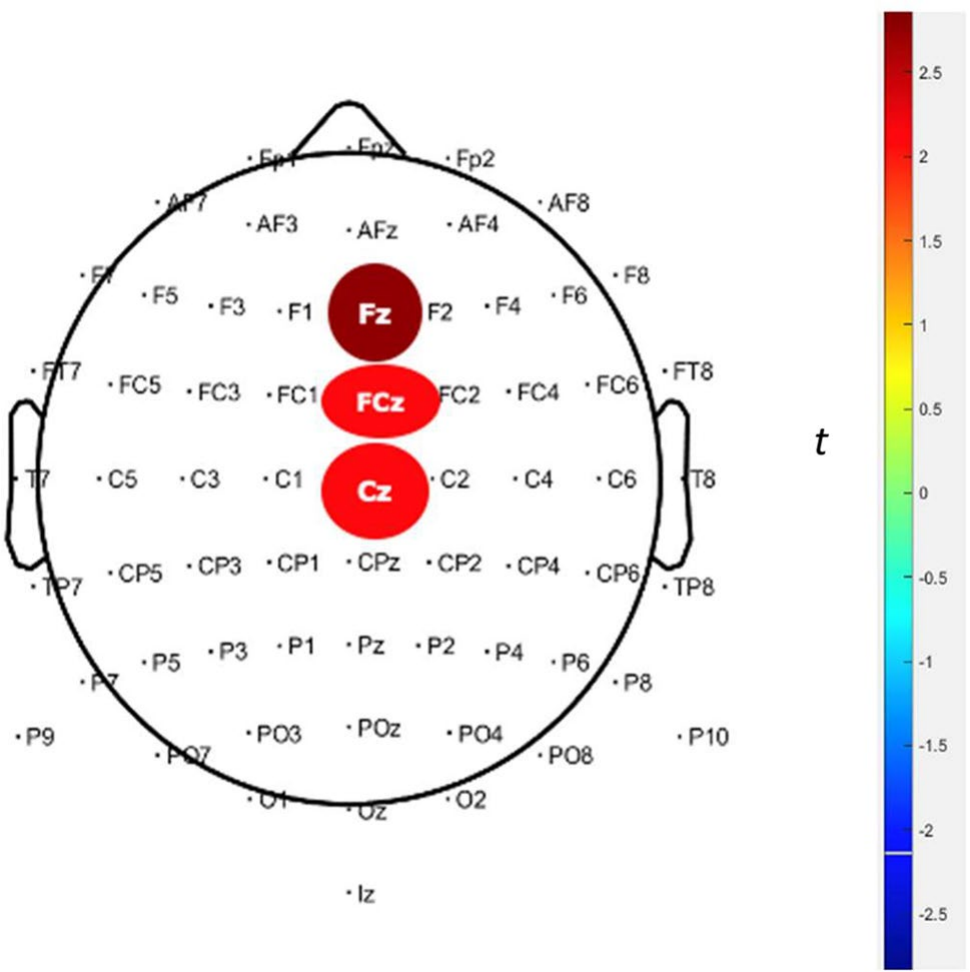
Results of the tmax permutation test on the amplitudes of the feedback-related negativity (FRN) between males in the humor group (MHg) and males in the nonhumor group (MNHg) during punishment feedback in the IGT. E-block four (trials 301–400). Time window 100–250 ms. 5,000 permutations. The spatial distribution of statistically significant electrodes in the difference between MHg and MNHg, where MHg showed a significant decrease in amplitude across all evaluated electrodes (FZ, FCZ, and CZ) compared with MNHg. *t* values vary from − 3 (blue) to 3 (red)

We were interested in sex-related effects of humor on FRN amplitude components differences, particularly during the final E-blocks (decision-making under risk). Figures 2 and 3 display representative waveform data and tmax permutation results, respectively, from E-block four during punishments, which helps to illustrate the essential cognitive control processes involved to implement corrective measures after experiencing punishments.

Upon examining FRN amplitude differences between females in the Hg and females in the NHg, no statistically significant differences were found during either reward or punishment scenarios. Comparing males and females in the Hg, it was observed that, during reward feedback, males exhibited a considerable reduction in FRN amplitude compared with females during E-block two (trials 101–200) at electrode FZ; then during E-block three (trials 201–300) and E-block four (301–400) at the three electrodes measured, during E-block five (trials 401–500) at electrodes FZ and FCZ. Additionally, during punishment feedback, males in the Hg displayed a decreased FRN amplitude compared with females in the Hg: in E-block one (trials 1–100) at electrode FZ, FCZ, and CZ; in E-block three at electrode FZ; in E-block four a decrease in amplitude in the three electrodes measured; and in E-block five at electrodes FZ and FCZ. Lastly, in assessing differences between males and females within the nonhumor context (males in the NHg and females in the NHg), no statistically significant differences were identified during either reward or punishment delivery.

### Differences in P3b

To identify clusters of adjacent values across electrodes and timepoints, we employed a series of cluster mass permutation tests. We applied an average difference waveform within the time interval of 350–450 ms, encompassing 36 electrodes, which was identified through the permutation analysis: FP1, AF7, F7, F5, FT7, C1, C3, C5, T7, CP1, CP3, TP7, P1, P3, P5, PO3, P9, FCZ, CZ, CPZ, PZ, POZ, OZ, C2, C4, T8, CP2, CP4, CP6, TP8, P2, P4, P6, P8, PO4, PO8. Within this framework, sex and group were considered as intergroup variables, whereas Type of Feedback (monetary reward and monetary punishment) and EEG-blocks [E-block one (trials 1–100), E-block two (trials 101–200), E-block three (trials 201–300), E-block four (trials 301–400), and E-block five (trials 401–500)] were regarded as intraparticipant factors. A comprehensive summary of the findings from the cluster permutation tests on P3b component variations is presented in Table 3.

**Table 3:** Results for cluster permutation test differences on IGT feedback (reward and punishment), for group (humor and nonhumor), sex (male and female), time window (350–450 ms), blocks (1–5), and electrodes (FP1–PO8)

Comparison	Type of feedback	Block	Trials IGT	Time window	Cluster	Electrodes	*t*	*p*
MHg-MNHg	Reward	1	1–100	350–450	1	P2	4.26	**.03**
		2	101–200	350–450	1	P2	2.8	**.03**
		3	201–300	350–450	1	P2	3.02	**.04**
		4	301–400	350–450	0	0	0	.05 *(NS)*
		5	401–500	350–450	1	P2	3.22	**.03**
	Punishment	1	1–100	350–450	1	P2	2.71	**.04**
		2	101–200	350–450	1	P2	2.71	**.04**
		3	201–300	350–450	1	P2	3.0	**.04**
		4	301–400	350–450	0	0	0	.05 *(NS)*
		5	401–500	350–450	1	P2	3.62	**.03**
FHg-FNHg	Reward	1	1–100	350–450	4	Fp1, AF7, FT7, C5, T7, TP7, P9, and P2	2.6	**[.03, .01]**
		2	101–200	350–450	2	Fp1, AF7, and P9	2.61	**[.04, .01]**
		3	201–300	350–450	4	Fp1, AF7, T7, P9, and P2	2.71	**[.04, .01]**
		4	301–400	350–450	3	Fp1, AF7, and P9	3.03	**[.03, .02]**
		5	401–500	350–450	1	Fp1 and AF7	4.2	**.01**
	Punishment	1	1–100	350–450	3	Fp1, AF7, FT7, C5, T7, and P9	2.68	**[.03, .01]**
		2	101–200	350–450	6	Fp1, AF7, F7, C1, C5, T7, CP1, TP7, P9, C2, C4, and P2	2.47	**[.04, .01]**
		3	201–300	350–450	3	Fp1, AF7, C5, T7, and P9	2.99	**[.02, .01]**
		4	301–400	350–450	5	Fp1, AF7, FT7, C1, C5, T7, CP1, CP3, TP7, P3, P5, P9, Pz, and P2	2.48	**[.03, .01]**
		5	401–500	350–450	2	Fp1, AF7, and T7	2.81	**[.04, .01]**
MHg-FHg	Reward	1	1–100	350–450	0	0	0	1.0 *(NS)*
		2	101–200	350–450	0	0	0	1.0 *(NS)*
		3	201–300	350–450	0	0	0	1.0 *(NS)*
		4	301–400	350–450	0	0	0	1.0 *(NS)*
		5	401–500	350–450	0	0	0	1.0 *(NS)*
	Punishment	1	1–100	350–450	0	0	0	1.0 *(NS)*
		2	101–200	350–450	0	0	0	1.0 *(NS)*
		3	201–300	350–450	0	0	0	1.0 *(NS)*
		4	301–400	350–450	0	0	0	1.0 *(NS)*
		5	401–500	350–450	0	0	0	1.0 *(NS)*
MNHg-FNHg	Reward	1	1–100	350–450	0	0	0	1.0 *(NS)*
		2	101–200	350–450	0	0	0	1.0 *(NS)*
		3	201–300	350–450	0	0	0	1.0 *(NS)*
		4	301–400	350–450	0	0	0	1.0 *(NS)*
		5	401–500	350–450	0	0	0	1.0 *(NS)*
	Punishment	1	1–100	350–450	0	0	0	1.0 *(NS)*
		2	101–200	350–450	0	0	0	1.0 *(NS)*
		3	201–300	350–450	0	0	0	1.0 *(NS)*
		4	301–400	350–450	0	0	0	1.0 *(NS)*
		5	401–500	350–450	0	0	0	1.0 *(NS)*

Cluster permutation tests results for P3 amplitude component during humor and nonhumor, across sex, and in response to reward and punishment feedback are as follows (5,000 permutations): MHg = male in humor group, MNHg = male in non-humor group, FHg = female in humor group, and FNHg = female in nonhumor group

Significant *t*-scores (*t*) and corrected *p*-values (*p*) within the [higher and lower *p* range value] range. Max distance between adjacent electrodes corresponds to approximately 3.88 cm (assuming a 56-cm head circumference)

**Fig. 4:**
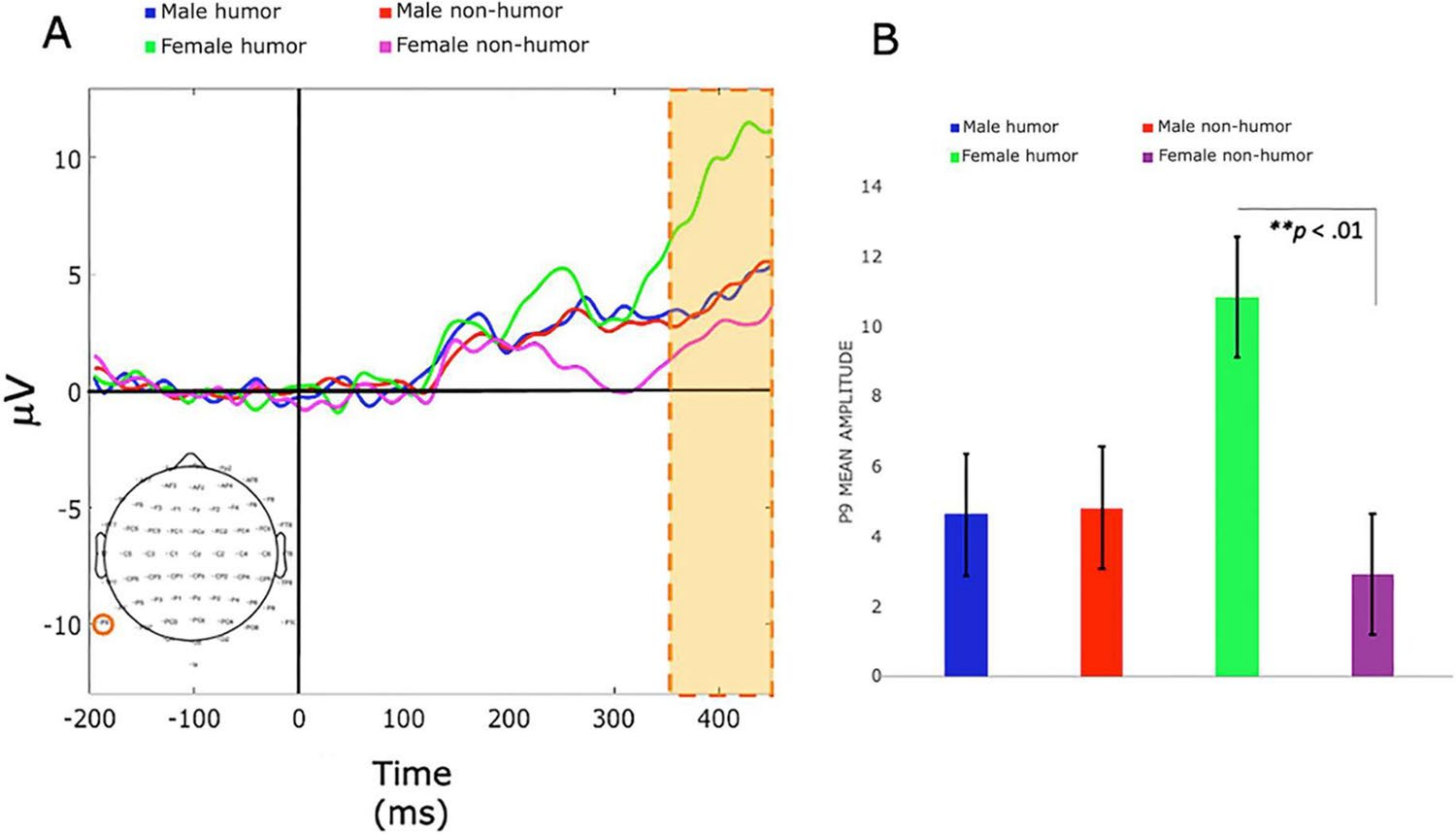
Grand-averaged ERP P3b waveforms from a representative electrode (P9) after punishment feedback for all groups. E-block four (trials 301–400) time window 350–450 ms. **A** Wave plot reveals statistically significant amplitude increases for electrode P9 in females in the Hg compared with females in the NHg. **B** Bar plot for P9 amplitude mean shows a significant increase in mean amplitudes for females in the Hg compared with females in the NHg. Error bars represent the standard error of the mean (SEM), and the yellow bar indicates statistically significant results within the time window measured

**Fig. 5:**
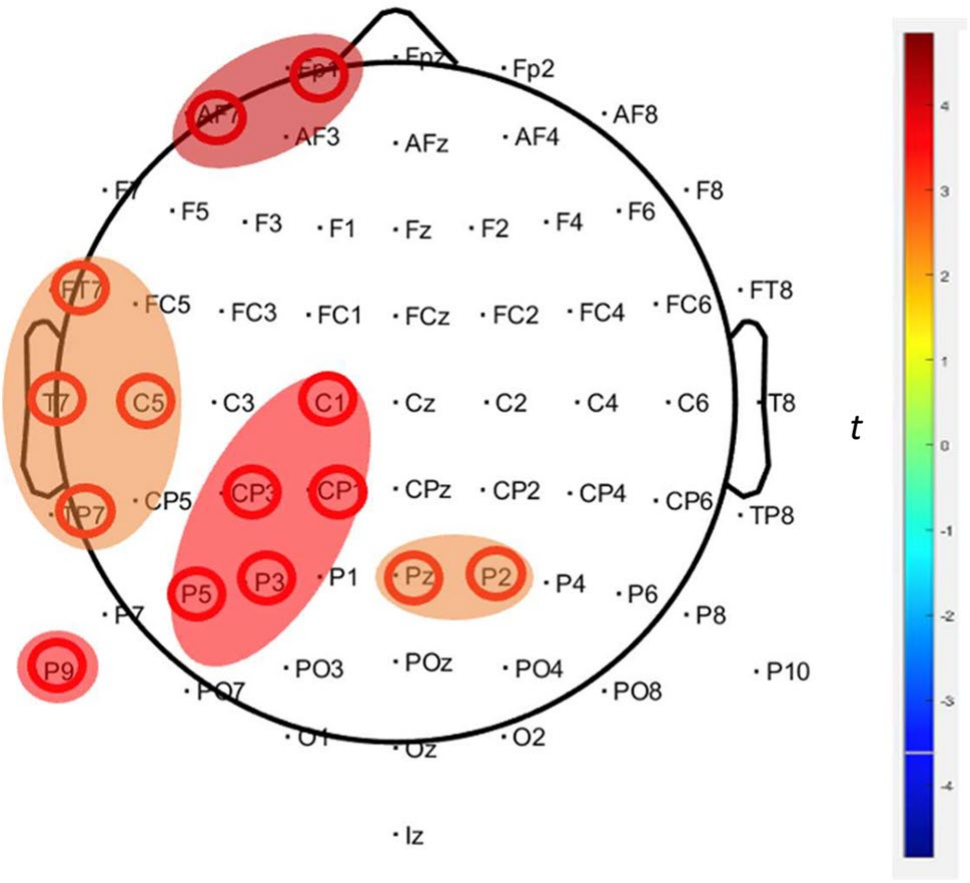
Cluster permutation test results for ERP P3b amplitudes between females in the Hg and females in the NHg during IGT punishment feedback (E-block four, trials 301–400). Time window 350–450 ms (5,000 permutations) as follows: the spatial distribution of five significant clusters (frontal-left, temporal-left, parietal-left, parietal-central, and parietal-right), resulting from the women in the Hg and NHg difference. *t*-values range from − 5 (blue) to 5 (red)

As summarized in Table 3, significant P3b amplitude differences were observed when comparing the males in the Hg with the males in the NHg. Contrary to our expectations, the males in the Hg exhibited increased P3b amplitudes during reward feedback compared with males in the NHg across E-block one (trials 1–100), E-block two (101–200), E-block three (201–300), and E-block five (trials 401–500), with a distinct cluster. Similar patterns were seen in the case of punishment, where males in the Hg consistently showed elevated P3b amplitudes compared with males in the NHg across E-blocks one, two, three, and five, again pinpointing the same cluster. Upon examining P3b amplitude disparities between females in the Hg and females in the NHg, and in line with our expectations, females in the Hg exhibited increased and highly distributed P3 amplitude activity during reward feedback compared with females in the NHg across all E-blocks; with four clusters in E-block one, two clusters in E-block two, four clusters in E-block three, three clusters in E-block four, and one cluster in E-block five. During punishment feedback, we also identified distributed increases in P3b amplitude activity across all E-blocks; with three clusters in E-block one, six clusters in E-block two, three clusters in E-block three, five clusters in E-block four, and two clusters in E-block five. Figures 4 and 5 display representative waveform data and cluster permutation results from E-block four during punishment. Therefore, E-block four serves to illustrate how cognitive control is allocating attentional resources to IGT performance when experiencing negative emotions.

Comparing males and females within the Hg, no statistically significant differences during either reward or punishment feedback were observed. Lastly, in assessing differences between males and females within the nonhumor group, no statistically significant differences were identified during either reward or punishment situations.

## Mediational analysis results

### Are the FRN amplitudes mediating the impairing effect of humor on IGT performance observed in males at IGT block six?

We conducted a path analysis by using only the sample of males to examine whether cognitive control, specifically FRN amplitudes, was mediating the impairing effect of humor on the IGT performance in males in Hg compared with males in the NHg at IGT block six. We considered humor as the independent variable, IGT performance during block six (IGT trials 126–150) as the dependent variable, and the mean of three electrodes (FZ, FCZ, and CZ) assessing FRN during reward on trial 1 to 100 as the mediator, because we had previously found differences in FRN amplitudes between males in the Hg and males in the NHg in these electrodes and trials. The indirect effect of humor on IGT performance through the FRN amplitudes was statistically significant (*IE* = 0.41; *SE* = 0.23; 95% CI = 0.08, 0.8). The direct effect of humor was not statistically significant (*DE* = 0.48; *SE* = 0.43; 95% CI =  − 0.22, 1.21). Therefore, the FRN amplitudes during rewards were fully mediating the impairing effect of humor observed at block six in males.

### Are the P3b amplitudes mediating the beneficial effect of humor in males during IGT block 20?

We conducted two path analyses on the sample of males to examine whether P3b amplitudes mediated the beneficial effect of humor on IGT during block 20. Humor was treated as the independent variable in both analyses, with IGT performance during block 20 (trials 476–500) as the dependent variable. In the first analysis, we considered the mean amplitude of electrode P2 assessing the P3b component during trials 201–300 in response to reward as the mediator, because we had previously found differences in P3b amplitudes between males in the Hg and males in the NHg in this particular electrode and trials. In the second analysis, the mean amplitude of electrode P2 in response to punishment during trials 201–300 was considered as the mediator.

Results for the first path analysis indicated that the indirect effect of humor on IGT performance through P3b amplitudes in response to rewards, was not statistically significant (*IE* = 0.11; *SE* = 0.23; *CI* 95% = -0.26, 0.48). Therefore, P3b amplitudes during rewards did not mediate the observed beneficial effect of humor during IGT block 20, among males. Similarly, results from the second path analysis did not reveal a statistically significant indirect effect of humor on IGT performance in response to punishment (*IE* =  − 5.43; *SE* = 10.93; 95% CI =  − 25.32, 8.87). Consequently, P3b amplitudes during punishments did not mediate the observed beneficial effect of humor during blocks 20, among males.

### Are the P3b amplitudes mediating the beneficial effect of humor on IGT performance observed in females during IGT blocks 19 and 20?

We conducted two path analyses by using exclusively the sample of females to examine whether P3b amplitudes mediated the beneficial effect of humor on IGT performance among females in the Hg compared with females in the NHg during blocks 19 and 20. Humor was treated as the independent variable in both analyses, with IGT performance during blocks 19 and 20 (trials 451–500) as the dependent variable. In the first analysis, we considered the mean amplitude of three electrodes (FP1, AF7, and P9) assessing the P3b component during trials 301–400 in response to reward as the mediator, because we had previously found differences in P3b amplitudes between females in the Hg and females in the NHg in these electrodes and trials. In the second analysis, the mean amplitude of 14 electrodes (FP1, AF7, FT7, C1, C5, T7, CP1, CP3, TP7, P3, P5, P9, Pz, and P2) in response to punishment was considered as the mediator.

Results for the first path analysis indicated that the indirect effect of humor on IGT performance through P3b amplitudes in response to rewards was not statistically significant (*IE* =  − 0.06; *SE* = 0.12; 95% CI =  − 0.3, 0.06). Therefore, P3b amplitudes during rewards did not mediate the observed beneficial effect of humor during IGT blocks 19–20, in females. Similarly, results from the second path analysis did not reveal a statistically significant indirect effect of humor on IGT performance in response to punishment (*IE* = 0.13; *SE* = 0.21; 95% CI =  − 0.24, 0.46). Consequently, P3b amplitudes during punishments did not mediate the observed beneficial effect of humor during blocks 19–20 in females.

## Discussion

The fact that humor improved IGT performance in females using an extended version (500 trials) of the IGT is in line with the results of our previous study (Flores-Torres et al., 2019), in which we used the standard version (100 trials) of the IGT. Our results also are in line with the findings of our other study (Flores-Torres et al., 2022), where we found that cognitive reappraisal—an emotion regulation strategy that increases positive emotion—also improved IGT decision-making in females. The present study extends our findings by showing that humor not only improved decision-making under risk in females but also in males increasing their cognitive control, specifically their attention allocation toward the task. However, the increase in cognitive control did not mediate the effect of humor on female’s IGT decision performance. We previously found Flores-Torres et al. (2022) that—in females—the influence of positive cognitive reappraisal on IGT decision-making was mediated by a reduction in negative emotions. Both a decrease in negative and/or an increase in positive emotions may be good alternative candidate mediators for the effect of humor on IGT performance. Unfortunately, we did not assess emotions in the present research. Future studies considering emotions as potential mediators of this relationship are needed.

The fact that humor had a positive effect during decision-making under risk (specifically at blocks 19 and 20) also in males’ contrasts with our previous findings. In our previous study, we found that humor impaired IGT decision making during the last block of the task. The discrepancy between the current and previous studies may be attributed to the fact that in the previous one, participants conducted fewer trials (100 trials); therefore, we were not able to observe the beneficial effect of humor in males. We were only able to observe the early distracting and impairing effect of humor during decision-making under ambiguity, as the males in the humor group did not learn to perform the task, and as a result, they never took decisions under risk. We considered that they could have learned the task (and consequently, performed decisions under risk) if they would have been provided with more trials, as in the extended version. In fact, in the current study, with more trials available (500 trials) humor was beneficial for decision-making under risk among males.

In the present study, we also found a detrimental effect of humor among men during decision making under ambiguity (specifically at block six), similar to the one above described found in the previous study with the standard IGT version (Flores-Torres et al., 2019). Specifically, during trials 126 to 150, males exposed to humor exhibited poorer performance than males not exposed to humor. This detrimental effect was not found among females. Furthermore, we found a significant reduction in task monitoring during rewards that mediated the detrimental impact of humor among males during trials 126–150. This reduction in monitoring serves as the mechanism through which humor decreases IGT decision-making during ambiguity. Hence, our findings evidenced our initial assumption that humor would demand greater cognitive and attentional resources among males than among females; however, it is essential to note that this negative effect is temporary.

Unexpectedly, humor increased males’ attention allocation toward the task (P3b amplitude); nonetheless, these increases in attention allocation did not mediate the beneficial effect of humor on their IGT performance under risk. In fact, the positive influence of humor on performance in men contrasts with the result of Flores-Torres et al. (2022), where we observed that another emotion-regulation strategy (i.e., cognitive reappraisal) impaired IGT decision-making for males. The differences between humor and reappraisal suggests that humor may not predominantly operate as a cognitive process in males (as in reappraisal) and opens the possibility of a beneficial effect of humor driven by increased motivation to tolerate fatigue (Polich, 2007; Varazzani et al., 2015). However, this hypothesis remains to be tested. Previous studies have indicated that the amplitude of P3b could be associated with an increase in norepinephrine and dopamine activity (Polich, 2007), which may in turn promote attention and memory retrieval, and decrease fatigue, which may serve to maintain the focus of attention on a task despite tiredness or boredom (Varazzani et al., 2015).

We also observed a more intense and highly distributed effect of humor in P3b component among females. The increased amplitude observed, especially during punishments compared with females in the nonhumor group, also was lateralized such that left electrodes (from frontal to parietal sites) showed a strong activation. These findings are in line with Cunningham et al. (2005) and Cui et al. (2013), who suggest that according to the emotional asymmetry hypothesis, positive emotion is evoked more strongly in the left hemisphere, which also is more sensitive to reward learning and positive situations, which are reflected in an increase in left lateralized amplitude in P3b activity. Our findings suggest that females in the humor group, during trials 350–400, may have experienced more positive emotions as a result of humor induction, compared with females in the nonhumor condition, who were not exposed to humor. Therefore, during punishment feedback, they successfully signaled their choices and accordingly, changed their strategy to improve their performance. Additionally, during humor, females had larger P3b amplitudes than males; specifically, they showed an increase in bilateral parietal sites, which reflects comparatively better attention and working memory than males (Horowitz-Kraus, 2014).

The present study has some limitations. We did not measure subjective positive and negative emotions during the IGT. Future studies should consider including self-report measures, such as the PANAS to assess affect. Additionally, our study only considered sex as a binary variable, and it is recommended that future studies measure both—sex assigned at birth and gender identity—and incorporate a broader range of gender identities. It also is important to control for the potential influence of task-switching costs, because this variable may interact with sex. Some of our analyses may have lacked sufficient statistical power owing to the limited number of participants and conditions. For example, contrary to our expectations, we did not find a significant effect of humor in blocks 17 and 18, although we observed trend-level findings. It is imperative for future studies to replicate these analyses with a larger sample size. Finally, our sample comprised only undergraduate college students from several universities in Chile and may not generalize to other populations. Despite these limitations, our study has several important strengths. It partially replicates a previous study on the impact of humor on the IGT and provides insights into the cognitive and electrophysiological mechanisms underlying sex-specific effects. Importantly, this is the first study to identify a mechanism explaining that males exposed to humor at the beginning of the task experience a decrease in their monitoring ability, especially after reward feedback, which impaired their learning. Unlike previous studies focusing solely on emotional effects, our research considers both sex and humor, offering a more comprehensive understanding of decision-making. Furthermore, we employed rigorous statistical corrections, ensuring the reliability and validity of our behavioral and EEG findings.

## Conclusions

Humor benefits decision-making under risk in both females and males (specifically at blocks 19 and 20) and attention allocation for most IGT blocks (P3b amplitudes). However, humor impaired men’s IGT decision-making under ambiguity during the block six and task monitoring (FRN amplitudes) for most IGT blocks. Attention allocation increases did not mediate the beneficial effect of humor on decision-making under risk neither among females nor males. Nonetheless, task monitoring decrements fully mediated the humor's detrimental influence on men's decision-making under ambiguity during block six.

## Supplementary Information

Below is the link to the electronic supplementary material.

Supplementary file1 (DOCX 2492 kb)

## Footnotes

1 There is no consensus in the exact timing where this process begins and ends (Bechara 1997, 2000; Dunn et al., 2006; Schiebener et al., 2011).

2 In the present manuscript, we use the word “sex” as our measure included the options of male and female. However, we did not measure sex and gender separately. When discussing previous findings, we will use the terms used by authors of those papers. We acknowledge that in many cases, females identify as women and males identify as men, but that previous research (as well as our own present measure) excludes some experiences of gender.

## Declarations

### Data Availability

The datasets used in this study are available upon reasonable request. Researchers interested in accessing the data for replication, validation, or further investigation should contact the following email address of the corresponding author: lydiagomez@uma.es. We will share datasets in compliance with ethical standards, confidentiality requirements, and data protection regulations. The goal is to facilitate transparency, reproducibility, and the advancement of scientific knowledge. We will evaluate requests for data access to ensure that they align with the original research purpose and adhere to relevant ethical and legal considerations.
