# Supplementary material for: Enhancing cognitive control of our decisions: Making the most of humor during the IGT in females and males
Source: Cogn Affect Behav Neurosci. 2024 Sep 5;24(6):1031–47. doi: 10.3758/s13415-024-01210-y (PMC11525253; doi:10.3758/s13415-024-01210-y)
Supplement: Supplementary file 1 — Supplementary file1 (DOCX 2492 kb) [file 13415_2024_1210_MOESM1_ESM.docx]

Supplementary material

*I Humorous and non-humorous videos*

To induce humor, we selected 200 videos (100 humorous and 100 non-humorous) from 240 public access videos (120 humorous and 120 non-humorous) available at www.youtube.com. Selection criteria were the presence or absence of humor in ecological situations [i.e., humorous videos depicted situations with non-sensical or with incongruity resolution structure while non-humor videos depicted mundane situations in which nothing of any emotional impact occurred (e.g., mowing the lawn, walking down the street)], and an adequate duration of stimuli to present a video before each decision (raw video mean duration = 12.84 s; video SD = 5.81 s). The total 240 videos were presented in a randomized order to 50 subjects (25 men and 25 women) who were not part of the present sample. We asked them to rate the videos using a humor scale ranging from 0 to 10 points (0 “not humorous at all”; 10 “the most humorous thing ever”). We eliminated all the videos that showed significant differences in ratings between men and women, as well as those less than three standard deviations away from the mean of the opposite condition, which resulted in the elimination of 40 videos (20 humorous and 20 non-humorous). The final video selection consisted of 100 humorous and 100 non-humorous videos. Men (humor: mean = 4.18, SD = 0.51; non-humor: mean = 1.50, SD = 0.25). Women (humor: mean = 4.24, SD = 0.46; non-humor: mean = 1.51, SD = 0.23). To examine whether there were statistically significant differences in the video ratings, we conducted a two-way factorial ANOVA (Gender × Video type [humor/non-humor]). Results showed a main effect of video type (*F*_1,198_ = 220, *p* < 0.001, *η*^2^ = 0.917), indicating that humorous videos were rated as significantly more humorous than non-humorous videos. Neither the main effect of Gender nor the interaction (Gender × Video type) was statistically significant. Humorous videos were assigned to the experimental group and non-humorous videos to the control group. The mean duration of the final selection of the videos was 12.91 (SD = 5.89) and 10.41 s (SD = 5.03) for the humorous and the non-humorous videos, respectively. For examples of non-humor and humor situations see figures 1 and 2.

All videos used in the current study are available at the following link: <https://www.dropbox.com/sh/qc8n4f2j6v594xs/AABcN7cnh_W-QY3s9KCdpiRHa?dl=0>

Non experimental (non-humor video)


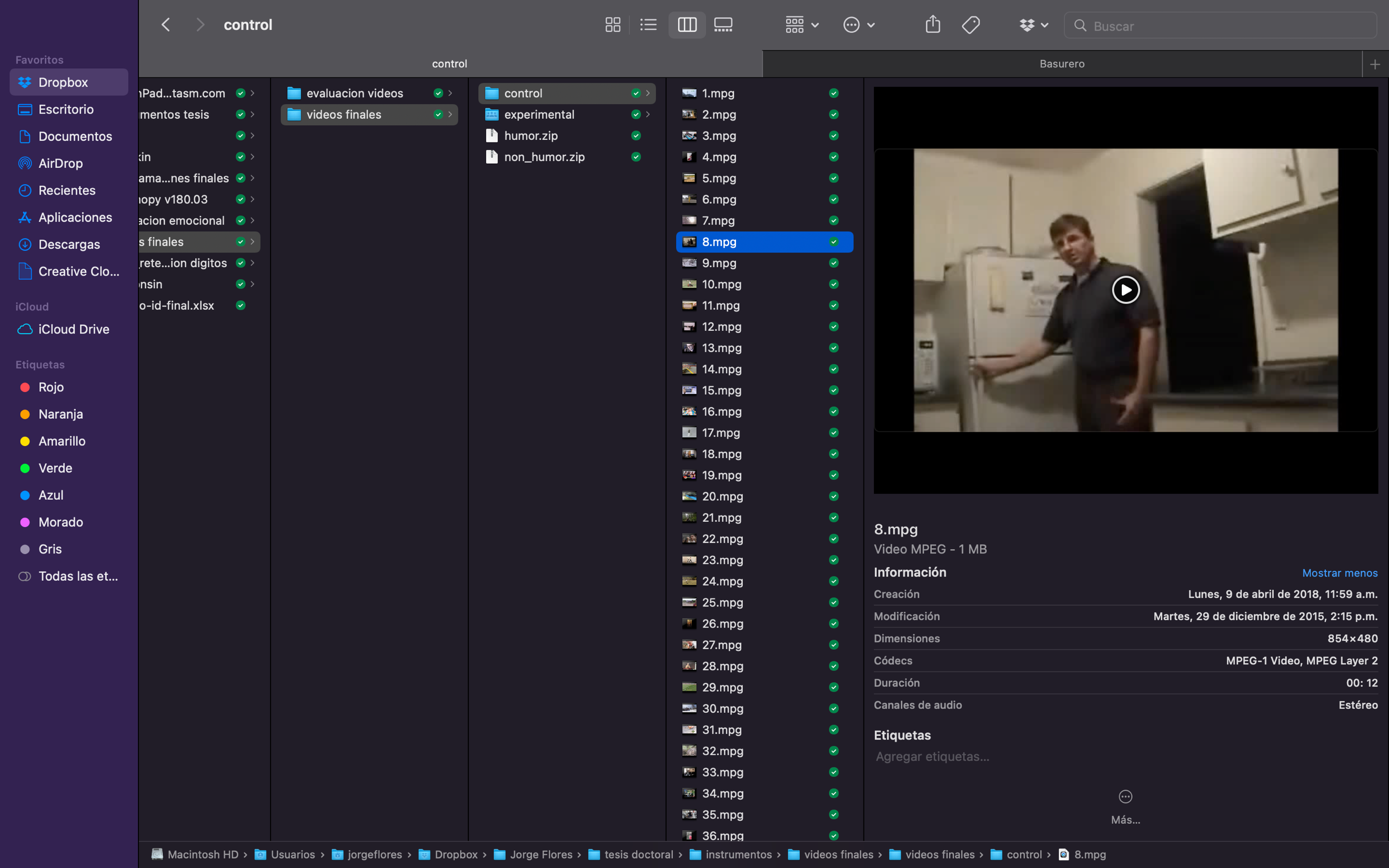


**Figure 1**: example of non-humorous video. The situation depicts a man showing how he fixed the door of his refrigerator (0.12 secs).

Experimental (humor video)


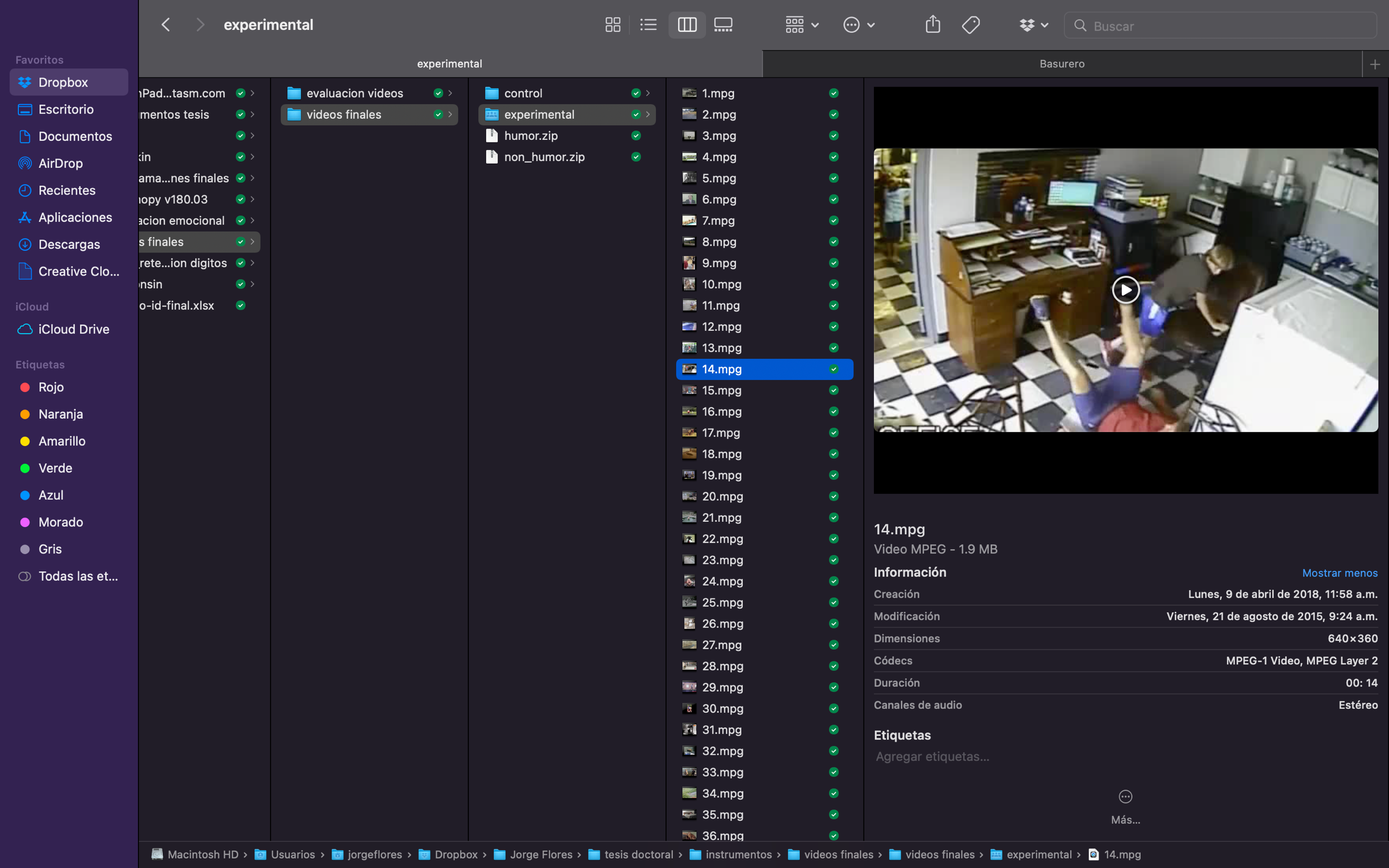


**Figure 2**: example of humorous video. The situation depicts a man falling after a boy, inadvertently, moved the man’s chair to open the refrigerator door at the back of the room (0.14 secs).
